# An Overview of Adalimumab Therapy for Ankylosing Spondylitis

**DOI:** 10.2174/0115733971289295240223095751

**Published:** 2024-02-26

**Authors:** Anna M. Sukhanova, Mariam A. Gilavian, Elizaveta V. Melnik, Evgenia V. Shikh, Alexey E. Petukhov, Vladimir I. Gegechkori, Sergey P. Dementev, Alexander M. Vlasov, Galina V. Ramenskaya

**Affiliations:** 1 Federal State Autonomous Educational Institution of Higher Education, I.M. Sechenov First Moscow State Medical University of the Ministry of Health of the Russian Federation (Sechenov University), 8-2 Trubetskaya Str., 119991 Moscow, Russia;; 2 Moscow Research and Practical Centre on Addictions of Moscow, Department of Public Health, 37/1 Lyublinskaya Str., 109390 Moscow, Russia

**Keywords:** Adalimumab, ankylosing spondylitis (AS), tumor necrosis factor (TNF), systematic review, efficacy, safety, randomized controlled trials

## Abstract

**Background:**

Ankylosing spondylitis (AS) is a chronic inflammatory disease known for causing pain, stiffness, and reduced mobility in the axial skeleton. Adalimumab, a tumor necrosis factor (TNF) inhibitor, has emerged as a promising therapeutic option for AS.

**Methods:**

This systematic review involved a comprehensive search of randomized controlled trials related to AS treatment, conducted in major databases such as MEDLINE, Google Scholar, and PubMed. The search terms encompassed ankylosing spondylitis, adalimumab, methotrexate, other non-biologic DMARDs, glucocorticoids, NSAIDs, and analgesics. A total of 14 randomized controlled trials with 4,500 participants were included in the review.

**Results:**

The review's results revealed that adalimumab demonstrated notable superiority when compared to a placebo. It effectively reduced disease activity, improved physical function, and lowered inflammatory markers such as C-reactive protein and erythrocyte sedimentation rate. Adalimumab demonstrated a favorable safety profile, with adverse events comparable to those observed with placebo.

**Conclusion:**

Based on the results, adalimumab is deemed an effective treatment for AS, showcasing its potential as a first-line therapeutic option. Notably, no significant increase in adverse events was observed compared to placebo. However, the conclusion emphasizes the need for further studies with extended follow-up durations to ascertain the long-term efficacy and safety of adalimumab in AS management. This systematic review provides valuable insights supporting the use of adalimumab in the treatment of AS and underscores the importance of ongoing investigations into its long-term effects to optimize its clinical utilization in AS patients.

## 1 INTRODUCTION

Ankylosing spondylitis (AS) is a chronic inflammatory disease that primarily affects the axial skeleton, causing pain, stiffness, and decreased mobility [1, 2]. Individuals with ankylosing spondylitis may experience changes in posture as the disease progresses, involving cervical flexion, reduced lumbar lordosis, posterior rotation of the pelvis, hip extension, knee flexion, ankle plantar flexion, and kyphosis [3]. AS often begins at a relatively young age, presenting a higher risk of hip complications [4, 5]. It is noteworthy that 40% of AS patients experience hip involvement [6]. Symptoms typically start to manifest between the ages of 15 and 25, with a higher prevalence in males [7, 8]. Approximately 30% to 50% of individuals with AS experience hip involvement, with 47% to 90% of those cases manifesting bilaterally [9-13]. Alongside hip issues, stiffness in the spine and intra-thoracic problems can contribute to significant disability in individuals with AS [9]. When painful degenerative changes affect the hip, studies have demonstrated that total hip arthroplasty (THA) can relieve pain and enhance function in individuals with ankylosing spondylitis (AS) [14-17]. However, performing THA in AS patients poses challenges due to various factors [18]. The procedure becomes complex because of the challenging approach and exposure of the hip caused by the presence of an ankylosed joint. Additionally, there is a risk of implant malposition attributed to sagittal plane malrotation of the pelvis [7] AS is classified within the spondyloarthritis (SpA) category, which constitutes a grouping of interrelated conditions distinguished by shared characteristics, including inflammatory back pain, asymmetrical oligoarthritis affecting the lower limbs, the potential evolution of enthesitis into ankylosis, and specific organ manifestations such as uveitis, bowel disease, psoriasis, and interstitial fibrosis in the lungs [19-21]. The cohort demonstrates a strong genetic correlation with the existence of the human leukocyte antigen B27 (HLA-B27) antigen [1, 22-24]. AS has also been termed radiographic axial spondyloarthritis (axSpA) and non-radiographic axSpA according to the new Assessment of SpondyloArthritis International Society (ASAS) criteria [25, 26]. AS has a significant impact on the quality of life of affected individuals, and its treatment can be challenging. Non-steroidal anti-inflammatory drugs (NSAIDs) are the first-line treatment option for AS patients, followed by conventional synthetic disease-modifying antirheumatic drugs (csDMARDs), including methotrexate, leflunomide, sulfasalazine, hydroxychloroquine, azathioprine, cyclosporine, cyclophosphamide, auranofin, penicillamine, and thalidomide, which are generally not effective in the treatment of axial manifestations of spondyloarthritis, but csDMARDs are effective for particular cases of peripheral AS [2, 27-30]. Over the past two decades, biologic agents, particularly tumor necrosis factor (TNF) inhibitors, have revolutionized the treatment of AS [31]. TNF inhibitors, such as adalimumab, have been shown to be effective in reducing disease activity, improving physical function, and reducing inflammation in patients with AS [32-36]. Adalimumab is a fully human monoclonal antibody that targets TNF-alpha and is also indicated for the treatment of six immune-mediated inflammatory diseases: rheumatoid arthritis (RA), juvenile idiopathic arthritis (JIA), ankylosing spondylitis (AS), psoriatic arthritis (PsA), psoriasis (Ps) and Crohn's disease (CD) [37-52]. It has been approved for the treatment of AS by regulatory authorities worldwide. While the use of adalimumab in the treatment of AS has been extensively studied in randomized controlled trials (RCTs), there is a need for a systematic review to evaluate the efficacy and safety of adalimumab in this population. This systematic review aims to provide an up-to-date and comprehensive evaluation of the current evidence on the use of adalimumab in the treatment of AS. The results of this systematic review can help inform clinical decision-making and guide the management of patients with AS.

## 2 MATERIALS AND METHODS

### 2.1 Methods

**Fig. (1):**
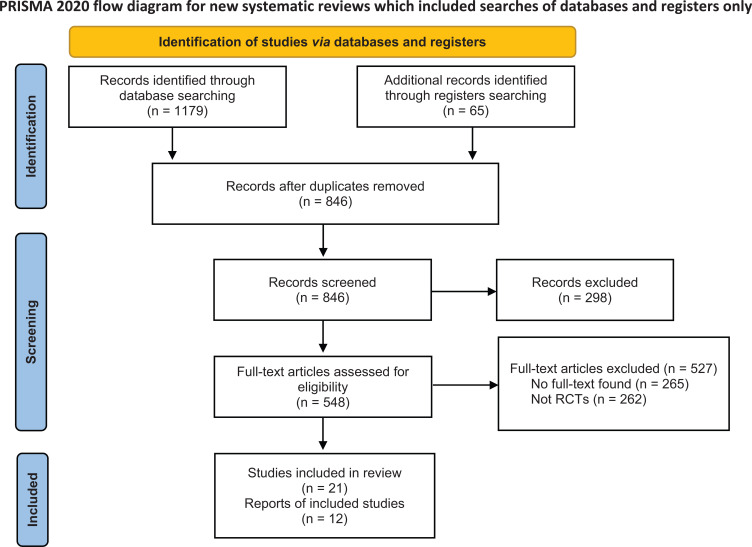
PRISMA Flowchart.

The Preferred Reporting Items for Systematic Reviews and Meta-Analyses (PRISMA) criteria were followed during the conduct of this systematic review and meta-analysis. A review protocol (CRD42023409768) was also registered in the International Prospective Register of Systematic Reviews, PROSPERO (Fig. **1**).

### 2.2 Search Strategy

A thorough exploration of the existing literature was performed to locate publications detailing randomized controlled trials (RCTs) assessing the effectiveness of adalimumab for treating ankylosing spondylitis. The search was performed using electronic databases, including Ovid MEDLINE, Embase, and The Cochrane Library, as well as clinical trial registries such as Clinicaltrials.gov and WHO trials registry (ICTRP), from inception up to January 9, 2023. The search strategy included a combination of MeSH headings and text words such as “adalimumab” (Humira) and “ankylosing spondylitis” (Bechterew disease or Marie-Strümpell disease). The search was limited to articles published in English from 2013 to March 2023. Gray literature and unpublished data were also searched. Recursive searches and cross-referencing were carried out, and the bibliography of identified articles was manually reviewed for additional relevant studies.

### 2.3 Inclusion and Exclusion Criteria

For this systematic review, randomized controlled trials assessing the efficacy and safety of adalimumab in the treatment of ankylosing spondylitis were considered for inclusion. Studies published as abstracts were enrolled only when full-text articles were available, and authors could be contacted for additional information on efficacy and safety outcomes. Participants older than 18 years diagnosed with ankylosing spondylitis were considered for inclusion. Participants must have had active ankylosing spondylitis at the study entry to be included. Studies that focused on other types of spondyloarthritis or inflammatory conditions were excluded. Interventions that involved adalimumab *versus* placebo or a control therapy were considered for inclusion.

### 2.4 The Outcome Measures used in the Studies to Evaluate the Effectiveness of Adalimumab

Typically, the evaluation of disease activity, physical function, and mobility involves the utilization of the Bath Ankylosing Spondylitis Disease Activity Index (BASDAI), Bath Ankylosing Spondylitis Functional Index (BASFI), and Bath Ankylosing Spondylitis Metrology Index (BASMI). Nevertheless, the ASAS (Assessment of SpondyloArthritis International Society) recommends the adoption of novel outcome measures in clinical trials focusing on symptom-modifying therapy. These include criteria such as ASAS 20 and ASAS 40 improvement, ASAS Partial Remission, and ASAS 5/6. The Assessment of SpondyloArthritis International Society (ASAS) has additionally confirmed the legitimacy of the Ankylosing Spondylitis Disease Activity Score (ASDAS). This scoring system incorporates C-reactive protein levels and certain inquiries from the Bath Ankylosing Spondylitis Disease Activity Index (BASDAI) to evaluate disease activity in individuals with ankylosing spondylitis (AS).

## 3 RESULTS

### 3.1 Pharmacology of Adalimumab

Adalimumab is a full-length, bivalent monoclonal antibody of the IgG1-κ class, weighing 150 kD. Its specific target is TNFα, which includes both soluble (sTNFα) and membrane-bound (mTNFα) forms [1].

The principal mode of action of adalimumab involves inhibiting the interaction between TNFα and its cell surface receptors, p55 and p75. It specifically targets soluble TNFα (sTNFα) molecules, which may form multimeric complexes, and membrane-bound TNFα (mTNFα) molecules capable of cross-linking and reversing intracellular signaling. By binding to these molecules, adalimumab prevents them from binding to natural TNFα receptors, neutralizing both sTNFα and mTNFα directly. Adalimumab also induces apoptosis and suppresses cytokines through reverse mTNFα-mediated signaling. Moreover, it has the potential to induce antibody-dependent cell-mediated cytotoxicity and complement-dependent cytotoxicity directed at cells that express membrane-bound TNFα (mTNFα). These various mechanisms contribute to the efficacy of adalimumab in the treatment of ankylosing spondylitis [1, 53, 54].

Following  the  subcutaneous  administration  of  a lone 40 mg dose of adalimumab in healthy adults, the drug undergoes gradual absorption, resulting in a peak serum concentration of 4.7 ± 1.6 µg/mL. This maximum concentration is attained approximately 131 ± 56 hours after administration. Adalimumab is primarily metabolized by proteolytic degradation, and the metabolites are eliminated through the kidneys and the feces. The average absolute bioavailability of the drug is 64% [1]. Based on research findings, the medication remains stable for a duration of 24 months when stored within the temperature range of 2°C to 8°C. Following the administration of a 40 mg dose, adalimumab exhibits a terminal half-life of approximately 2 weeks, falling within a range of 10 to 20 days. The total body clearance is reported to be in the range of 0.18-0.27 mL/minute [1].

### 3.2 Efficacy of Adalimumab in AS

#### 3.2.1 Clinical Trials Analyzing the Efficacy of Adalimumab in AS

Adalimumab underwent clinical trials starting in 1997, and it received approval from the US Food and Drug Administration (FDA) in 2006 for the treatment of ankylosing spondylitis (AS). Table **1** provides a summary of the main randomized, clinical trials that have examined the efficacy of adalimumab in AS.

Among these clinical trials, a pivotal investigation was the ATLAS trial (Adalimumab Trial Evaluating Long-Term Safety and Efficacy for Ankylosing Spondylitis). This trial comprised a 24-week, randomized, double-blind, and placebo-controlled study involving 315 patients with active ankylosing spondylitis (AS). Notably, the patients in this trial were managed concurrently with conventional treatment despite their ongoing active AS [1]. This trial juxtaposed adalimumab 40 mg administered every other week with a placebo. The primary efficacy measure focused on the percentage of patients achieving an Assessment in Ankylosing Spondylitis (ASAS) 20 response [1]. Results indicated that by the 12th week, a significantly higher proportion of patients treated with adalimumab achieved the primary endpoint compared to those on placebo (58% *versus* 21%, respectively; *P* = 0.001). Noteworthy was the early onset of therapeutic effects with adalimumab evident as early as week 2 (42% *versus* 16%; *P* = 0.001). Interestingly, even among patients with total spinal ankylosis, ASAS 20 responses were observed at week 12 (3 out of 6 patients *versus* 0 out of 5 patients), although statistical significance was not attained due to the limited sample size [1]. Adalimumab also exhibited efficacy in reducing enthesitis, as assessed by the Maastricht Ankylosing Spondylitis Enthesitis Score (2.7 *versus* 1.3; *P* = 0.02) [1]. The disparities in outcome scores were sustained throughout the entire duration of the trial (24 weeks) [1a].

In 2009, Rudwaleit *et al.* published an international, unblinded investigation examining the effectiveness of adalimumab therapy in 1,250 individuals with active ankylosing spondylitis (AS) spanning a 12-week duration. The study found that a significant proportion of patients achieved treatment responses based on various measures. Specifically, 57.2% of patients achieved a 50% improvement in the Bath Ankylosing Spondylitis Disease Activity Index (BASDAI 50), 53.7% achieved a 40% improvement according to the Assessment in Ankylosing Spondylitis (ASAS 40) criteria, and 27.7% attained ASAS Partial Remission (ASAS PR). The investigators pinpointed numerous factors exhibiting a robust correlation with favorable treatment responses. These factors encompassed a younger age, heightened levels of C-reactive protein (CRP), the presence of human leukocyte antigen HLA-B27, and being naïve to anti-tumor necrosis factor (TNF) therapy [1b].

In a randomized, double-blind, placebo-controlled trial conducted by Lambert *et al.* [1c], the efficacy of adalimumab in reducing spinal and sacroiliac (SI) joint inflammation was investigated using magnetic resonance imaging (MRI) in patients with active ankylosing spondylitis (AS). The extent of inflammation in the spine and SI joints was evaluated using the Spondyloarthritis Research Consortium of Canada (SPARCC) MRI index. The findings revealed a notable reduction in the spine SPARCC score among individuals administered adalimumab, contrasting with an increase observed in the placebo group (with a mean decrease of 53.6% *versus* a mean increase of 9.4% from baseline, respectively; *P* < 0.001). Similarly, the SI joint SPARCC score witnessed a significant decrease in both groups (with a mean decrease of 52.9% in the adalimumab group compared to 12.7% in the placebo group; *P* = 0.017). The positive response noted in the adalimumab group endured through week 52. Another randomized, controlled trial demonstrated significant improvement in MRI inflammation measurements, with a notable decrease in lumbar spine and sacroiliac (SI) joint SPARCC scores in patients with active ankylosing spondylitis (AS) who received adalimumab treatment for 12 weeks [1]. Moreover, an alternate open-label investigation employed a semiquantitative scoring system (ranging from 0 to 12) to evaluate inflammation in the sacroiliac (SI) joints among individuals with ankylosing spondylitis (AS) undergoing adalimumab treatment. Nevertheless, this study did not uncover a substantial alteration in MRI scores in contrast to the baseline, a circumstance potentially ascribed to the small sample size and/or the limited responsiveness of the employed scoring system [1].

In a randomized, controlled trial led by Huang *et al.*, the effectiveness of adalimumab was assessed in a group of 344 Chinese individuals diagnosed with ankylosing spondylitis (AS) [55]. Following 12 weeks of treatment, adalimumab displayed a notably higher percentage of ASAS 20 responders compared to the placebo group (67.2% *versus* 30.4%, respectively; *P* < 0.001). Moreover, adalimumab demonstrated superior outcomes in ASAS 40 response, ASAS 5/6 response, and the attainment of ASDAS inactive disease status at week 12, with statistically significant distinctions observed between the adalimumab and placebo groups (*P* < 0.001 for all comparisons) [55].

Overall, these clinical trials have provided robust evidence supporting the efficacy of adalimumab in the treatment of AS, with positive outcomes observed in terms of disease activity, function, enthesitis, and inflammation.

#### 3.2.2 Effectiveness in Non-Articular Manifestations of Ankylosing Spondylitis

The effectiveness of adalimumab in treating extra-articular manifestations associated with ankylosing spondylitis (AS) has been extensively studied, demonstrating its efficacy in managing these manifestations and providing comprehensive therapeutic benefits to patients.

Uveitis, inflammation of the uveal tract of the eye, is a common extra-articular manifestation in AS. Clinical trials have consistently shown that adalimumab treatment leads to a significant reduction in the frequency and severity of uveitis flares in AS patients [1]. A study conducted in an open-label setting found that adalimumab showed a potential reduction in flare rates by approximately 50% in a cohort of patients with ankylosing spondylitis (AS) comprising 1,250 individuals [1].

Enthesitis, characterized by inflammation at the insertion sites, where tendons, ligaments, capsules, fascia, and bones meet [55, 56], is another prevalent extra-articular manifestation in AS. Adalimumab has been demonstrated to effectively reduce enthesitis-related symptoms, including pain and swelling while improving overall physical function in AS patients. Validated scoring systems, such as the Maastricht Ankylosing Spondylitis Enthesitis Score, have been utilized to assess the efficacy of adalimumab in managing enthesitis [1].

Adalimumab has also shown efficacy in addressing other extra-articular manifestations, such as psoriasis and inflammatory bowel disease (IBD). In AS patients with concurrent psoriasis, adalimumab treatment has resulted in symptom improvement and management of psoriatic skin lesions [1]. Similarly, in AS patients with coexisting IBD, adalimumab has exhibited positive clinical outcomes by reducing disease activity and promoting healing of the intestinal mucosa [1].

Furthermore, it is pertinent to acknowledge the therapeutic efficacy of Adalimumab in managing degenerative arthritis of the hip joint and other appendicular joints that frequently accompany AS. Extending the discussion to encompass these appendicular joint involvements broadens our understanding of the holistic benefits offered by Adalimumab in addressing the diverse spectrum of AS manifestations.

#### 3.2.3 Effectiveness of Adalimumab in Treating Non-Radiographic Axial Spondyloarthritis

Axial spondyloarthritis primarily affects the axial skeleton and is characterized by inflammatory arthritis [57]. While ankylosing spondylitis (AS) shows characteristic changes in the sacroiliac joints on plain radiography, non-radiographic axial spondyloarthritis (nr-axSpA) lacks such definitive radiographic evidence [57]. To classify nr-axSpA, the SpondyloArthritis International Society (ASAS) developed criteria that include objective findings and exclude other possible causes of back pain [58, 59]. The diagnosis and treatment of nr-axSpA can be challenging due to its heterogeneous presentation and lack of a diagnostic biomarker.

Clinical trials have evaluated the efficacy of adalimumab in nr-axSpA. The ABILITY-1 study, conducted in patients with axial spondyloarthritis, demonstrated the effectiveness of adalimumab in nr-axSpA [60]. Adalimumab-treated patients showed significantly higher response rates based on ASAS 20 and ASAS 40 criteria compared to placebo at week 12. Adalimumab also showed significant efficacy based on other composite measures, such as ASAS 70, ASAS 5/6, BASDAI 50, ASDAS, and clinical remission [60]. Significantly, enhancements in quantifiable indicators of inflammation, such as CRP levels and SPARCC MRI scores for both sacroiliac joints and the spine, were noted with the administration of adalimumab [60].

#### 3.2.4 Extended Use of Adalimumab: Ensuring Long-Term Efficacy and Maintenance of Improvements in Ankylosing Spondylitis Patients

Managing a chronic disease like ankylosing spondylitis (AS) requires ensuring the long-term safety and effectiveness of treatment agents. Numerous studies have shown that TNFα antagonists, such as adalimumab, maintain their efficacy and safety over years of use in AS patients. A 5-year open-label follow-up study involving 125 AS patients who received adalimumab revealed that significant proportions of them achieved favorable outcomes: 70% reached ASAS40, 77% achieved BASDAI 50, 51% attained ASAS partial remission, and 61% reached ASDAS inactive disease [1]. Moreover, findings from a randomized controlled trial in non-radiographic axial spondyloarthritis indicated a positive correlation between younger age and favorable clinical outcomes following one year of adalimumab treatment [1]. However, it is crucial to note that antibodies against TNFα inhibitors can develop, leading to reduced efficacy and early discontinuation of anti-TNF therapy. In a study by Arends *et al.*, antibodies were detected in 30% of AS patients receiving adalimumab [1d]. Moving beyond antibody formation, a comprehensive evaluation of the long-term effects of adalimumab on AS patients was conducted. Patients received blinded adalimumab or placebo for 24 weeks, followed by open-label adalimumab for up to 5 years [61]. The study assessed spinal mobility using BASMIlin, as well as other clinical, functional, and AS quality-of-life outcomes. The results demonstrated that improvements in BASMIlin were sustained throughout the 5-year period, with significant correlations between BASMIlin and all evaluated clinical outcomes [61]. The strongest correlation was observed between BASMIlin and BASFI at both 12 weeks and 5 years [61]. Multivariate regression analysis further confirmed this association. Adalimumab treatment for up to 5 years in patients with active AS led to sustained benefits in spinal mobility, disease activity, physical function, and HRQoL [61].

### 3.3 Comparing the Efficacy of Adalimumab and Biosimilar Agents

Comparing the efficacy of adalimumab and biosimilar agents is crucial in addressing the limitations faced by patients with ankylosing spondylitis (AS) who require treatment with TNF-α inhibitors. Although TNF-α blockers have shown effectiveness in managing AS, their high cost poses a significant barrier, particularly for patients with modest salaries or inadequate healthcare insurance [62, 63]. This financial constraint has restricted access to these life-changing therapies for many individuals with AS who also experience extra-articular symptoms and have not responded to NSAIDs [63, 64]. Therefore, the comparison of adalimumab with biosimilar agents holds promise in potentially providing more affordable treatment options without compromising efficacy, ensuring that a broader population of AS patients can benefit from effective therapy.

#### 3.3.1 IBI303 *versus* Adalimumab

A phase 3 multicenter, double-blind, randomized controlled trial was conducted in China to compare the efficacy of IBI303, a biosimilar monoclonal antibody against TNFα, with adalimumab in the treatment of ankylosing spondylitis [65]. Biosimilars are typically developed once the patent protection of the reference biologic (in this case, Adalimumab) expires. This allows for the production of more affordable alternatives without infringing on intellectual property rights. The study involved 438 patients who were randomly assigned to receive either 40 mg of IBI303 or 40 mg of adalimumab as a subcutaneous injection every 2 weeks until week 22 [65]. The primary outcome measured was the proportion of patients achieving a 20% improvement according to the Assessment of SpondyloArthritis International Society (ASAS) Response Criteria at week 24 [65]. The results showed that in the full analysis population, 75% of patients in the IBI303 group and 72% in the adalimumab group reached the primary outcome, demonstrating therapeutic equivalence between the two drugs. Importantly, the 95% confidence intervals of the difference in responses between the two groups fell within the pre-specified equivalence boundaries of -15% to 15%, confirming the therapeutic equivalence of IBI303 and adalimumab [65]. The safety and tolerability profiles were also similar, with comparable rates of treatment-emergent adverse events. These findings suggest that IBI303 could serve as an alternative treatment option for Chinese patients with ankylosing spondylitis, providing a highly similar efficacy and safety profile to adalimumab.

#### 3.3.2 HS016 *versus* Adalimumab

A phase III clinical trial was carried out in China, employing a multicenter, randomized, double-blind, and parallel design to compare the efficacy and safety of the biosimilar candidate HS016 with adalimumab (Humira) for the treatment of active ankylosing spondylitis (AS) [2]. The study involved 648 patients with active AS who were randomly assigned to receive either HS016 or adalimumab subcutaneously every other week for 24 weeks [2]. The primary endpoint of achieving at least a 20% improvement (ASAS20) in patients at 24 weeks was similar between the HS016 group (87.5%) and the adalimumab group (90.1%) [2]. The difference in response rates fell within the predefined equivalence margin, indicating no significant difference in efficacy between the two treatments [2]. Secondary endpoints, encompassing alternative efficacy assessment parameters, health evaluations, safety measures, pharmacokinetics, and immunogenicity parameters, likewise demonstrated no notable distinctions. The rates of treatment-emergent adverse events were similar in both groups, with most being mild to moderate [2]. Plasma concentrations of HS016 and adalimumab were comparable, and the proportion of patients with positive human anti-human antibodies and neutralizing antibodies did not differ significantly between the groups [2]. Based on these findings, HS016 can be considered an affordable alternative for the treatment of Chinese patients with AS, providing similar efficacy and safety profiles to adalimumab.

The treatment of patients with AS has been significantly transformed in recent decades with the introduction of anti-TNFα medication [66]. However, the development of biologic agents has contributed to a rise in healthcare costs. Fortunately, the rapid development of biosimilars for drugs that are no longer under patent protection has provided affordable alternatives for patients and healthcare systems [67, 68]. These biosimilars offer comparable efficacy and safety profiles to the originator treatments. It has been estimated that the utilization of biosimilars could lead to a reduction of healthcare-related costs by US$54 billion from 2017 to 2026 in the United States alone [69]. Several investigations have examined the implementation of biosimilars, encompassing the transition from an originator treatment to a biosimilar [70-72], as well as the execution of direct comparisons between the originator and biosimilar [65]. This demonstrates the growing interest in harnessing the potential of biosimilars to enhance accessibility to effective treatments while addressing the economic challenges associated with biologic agents.

Additionally, a supplementary inquiry was conducted to evaluate the efficacy of HS016 and adalimumab within stratified subgroups across different time intervals. This assessment utilized the Health Assessment Questionnaire for Spondyloarthropathies (HAQ-S) and the short form 36 (SF-36) questionnaires as evaluative tools [73]. Individuals diagnosed with active ankylosing spondylitis (AS) were randomly allocated to receive subcutaneous injections of either 40 mg HS016 or adalimumab every two weeks for a duration of 24 weeks, following a 2:1 ratio. Mental and physical improvements were evaluated through health surveys and pertinent factors. The outcomes indicated that both HS016 and adalimumab demonstrated time-dependent enhancements in the Health Assessment Questionnaire for Spondyloarthropathies (HAQ-S) scores up to 14 weeks, with a swift decline observed during the initial 4 weeks of treatment. The Short Form-36 (SF-36) health survey revealed that both treatments exhibited prompt beneficial effects against AS within the initial 2 weeks, gradually diminishing between 2 and 12 weeks and stabilizing from 12 to 24 weeks. These findings imply that HS016 serves as an economically feasible alternative treatment for Chinese AS patients, providing rapid alleviation of symptoms and contributing to enhanced satisfaction with lifestyle.

### 3.4 The Comparative Effectiveness of Secukinumab and Adalimumab in Ankylosing Spondylitis

The study, conducted by Maksymowych *et al.*, assessed the comparative effectiveness of secukinumab 150 mg and adalimumab 40 mg in biologic-naïve patients with ankylosing spondylitis (AS) using a matching-adjusted indirect comparison method over a period of up to 1 year [74]. Pooled individual patient data from the secukinumab arms of the MEASURE 1 (NCT01358175) and MEASURE 2 (NCT01649375) trials (n=197) were matched with the adalimumab population from the ATLAS trial (NCT00085644) (n=208). Logistic regression analysis was utilized to determine weights for matching variables such as age, sex, Bath AS Functional Index, C-reactive protein levels, and previous tumor necrosis factor inhibitor therapy. Recalculated Assessment of SpondyloArthritis International Society (ASAS) 20 and 40 responses at various time points (weeks 8, 12, 16, 24, and 52) from the MEASURE 1/2 trials (effective sample size=120) were compared with those from the ATLAS trial. Placebo-anchored comparisons were feasible until week 12, while unanchored comparisons were necessary thereafter. Results indicated no differences between secukinumab and adalimumab in placebo-anchored ASAS 20 and 40 responses up to week 12. However, in unanchored comparisons, secukinumab demonstrated higher ASAS 20 response at week 16 (odds ratio 1.60 (95% confidence interval, 1.01-2.54); *p* = 0.047), higher ASAS 20 and 40 responses at week 24 (1.76 (1.11-2.79); *p* = 0.017 and 1.79 (1.14-2.82); *p* = 0.012, respectively), and higher ASAS 40 response at week 52 (1.54 (1.06-2.23); *p* = 0.023) compared to adalimumab. No differences were observed in placebo-adjusted ASAS 20 and 40 responses up to 12 weeks, but after week 12, secukinumab demonstrated greater improvement in non-placebo-adjusted ASAS 20 and 40 responses compared to adalimumab in patients with ankylosing spondylitis.

### 3.5 Successful Alleviation of Lumbar Radiculopathy in a Case of Ankylosing Spondylitis Through Adalimumab Therapy: A Study of a Single Patient

Ankylosing spondylitis (AS) is characterized by a worldwide prevalence ranging from 0.1% to 1.4%. It demonstrates a higher frequency of occurrence in males compared to females [75, 76]. Lumbar radiculopathy is a rare but debilitating manifestation of ankylosing spondylitis (AS), characterized by low back pain and radiating symptoms [75]. There is a single-patient case study highlighting the effective relief of lumbar radiculopathy in a 37-year-old male patient with AS through adalimumab treatment [75]. The patient had no significant past medical history but had undergone bilateral hip arthroplasty and bilateral rotator cuff repair surgery due to multiple motor vehicle accidents [75]. He initially presented with chronic lower back pain, rated as 8 out of 10 on the numerical rating scale (NRS) [75]. The pain was described as constant and aching, radiating from the lower back to both feet, accompanied by stiffness, numbness, and tingling [75]. Despite multiple interventions, including NSAIDs, opioids, trigger point injections, osteopathic manipulative treatment, and physical therapy, the patient experienced inadequate pain relief over a three-and-a-half-year period [75]. According to reports, ankylosing spondylitis (AS) shows a strong association with genetic variations in the PTGER4 gene [77]. This genetic variation has been linked to reduced responsiveness to nonsteroidal anti-inflammatory drugs (NSAIDs), as observed in the patient who exhibited poor response to multiple NSAIDs [77]. During the diagnostic journey, the patient developed bilateral eye redness and pain, prompting referral to an ophthalmologist and a rheumatologist [75]. Ankylosing spondylitis was diagnosed, and adalimumab, a tumor necrosis factor (TNF) blocking agent, was initiated [75]. Following the initiation of adalimumab, the patient experienced gradual improvement in low back pain, radicular leg pain, and bilateral sacroiliac joint pain, leading to a reduced need for pain medications [75]. In a follow-up visit one month later, the patient reported a lower pain intensity of 3-4/10 on the NRS, enabling him to return to work and resume his daily activities [75].

The timely initiation of tumor necrosis factor (TNF) blocking agents plays a critical role in the successful management of spondyloarthropathies by substantially reducing disease activity, leading to improved symptoms and radiographic sacroiliitis outcomes [77-79]. This case report demonstrates that the administration of adalimumab, a TNF-blocking agent, proved highly effective in alleviating chronic low back pain along with radicular symptoms, resulting in a reduced reliance on opioid medications [75].

### 3.6 Evaluating the Economic Impact, Quality of Life, and Occupational Outcomes of Adalimumab Treatment in Chinese Patients with Active Ankylosing Spondylitis

A prospective study was conducted in China involving 91 adult patients (aged ≥ 18 years) with active ankylosing spondylitis (AS) who met the 1984 New York modified criteria for AS and had a Bath Ankylosing Spondylitis Disease Activity Index (BASDAI) score ≥ 4 [80]. All participants received adalimumab treatment (40 mg every 2 weeks) and completed questionnaires regarding disease characteristics, quality of life, and cost. Work-related outcomes were assessed using the Work Limitation Questionnaire and the Work Productivity and Activity Impairment Questionnaire in AS, which were completed by patients with paid employment. Factors influencing work outcomes were analyzed.

The study included 91 patients with a mean age of 30 years (87.8% males) and a mean disease duration of 10 years who received adalimumab treatment for 24 weeks. The annual estimated cost per patient was $37,581.41, with the direct cost accounting for 84.6% of the total. Among the participants, 78% held paid employment, with an average work productivity loss of 0.28, according to the Work Limitation Questionnaire. Absenteeism and presenteeism rates were reported at 10.22% and 43.86%, respectively, resulting in a mean work productivity loss of 47.92% as assessed by the Work Productivity and Activity Impairment Questionnaire in ankylosing spondylitis (AS). Notably, subsequent to adalimumab treatment, significant enhancements were observed in presenteeism, absenteeism, work productivity, and overall quality of life.

The expenses incurred by ankylosing spondylitis (AS) patients undergoing adalimumab therapy were identified as substantial in China [80]. Adalimumab treatment resulted in substantial improvements in disease activity, physical function, quality of life, and work outcomes. Significantly, presenteeism was found to be associated with disease duration, BASFI score, and ASQoL score, while disease duration was linked to absenteeism. Age, education, BASFI score, and ASQoL score were identified as influencing factors in work productivity loss.

The variability in healthcare costs related to ankylosing spondylitis (AS) across different countries poses a challenge in comparing work-related costs beyond the country of origin [80]. A cohort study based on the population in Brazil disclosed that 78% of patients with ankylosing spondylitis (AS) initiated anti-TNF drug treatment at no charge, with a median monthly per capita cost of $1650 [81]. In the United Kingdom, a research study estimated the total annual cost of AS to be €19,016 per patient, with predominant expenses attributed to work-related costs [82]. In the study by Liudan Tu *et al.* [80], the average annual cost of illness per patient amounted to $37,581.41, primarily associated with adalimumab treatment. Considering China's GDP per capita of $8,800 in 2017, the overall annual cost borne by Chinese AS patients undergoing adalimumab treatment remained substantial [80]. Previous investigations conducted in Europe [83] and Australia [84] demonstrated a reduction in healthcare resource utilization and an increase in labor force participation rates following adalimumab therapy.

A European study demonstrated that after one year of adalimumab treatment, presenteeism dropped from 56.6% to 20.1%, absenteeism reduced from 15.6% to 6.4%, and total work productivity impairment decreased from 59.9% to 22.1% [85]. Various factors, including age [86], ethnicity [87], disease duration, disease activity [88, 89], physical function [89, 90], and quality of life [91], have been reported in other studies to significantly influence work outcomes.

Consistent with research conducted in Western [88, 90] and Asian populations [89], the study by Tu *et al.* [80] found that disease duration, BASFI, and ASQoL were linked to work outcomes. Intriguingly, no significant distinction in work outcomes was observed based on disease activity measures such as BASDAI or ASDAS, contrary to findings from other studies [88, 89]. Nonetheless, it remains crucial to manage disease activity to enhance work outcomes in individuals with ankylosing spondylitis (AS).

In light of the comprehensive data on adalimumab's effectiveness and economic impact, it is crucial to emphasize that while the cost of the medication is indeed a significant consideration, its role in improving work productivity should not be viewed in isolation. The findings from routine clinical practice in Central and Eastern European (CEE) countries indicate clinically meaningful improvements in disease activity, physical function, and substantial reductions in healthcare resource utilization and sick leave with originator adalimumab treatment [83]. Economic assessments conducted in the UK further support the cost-effectiveness of adalimumab and other anti-TNF agents compared with conventional therapy for treating patients with ankylosing spondylitis (AS) and non-radiographic axial spondyloarthritis (SpA) [83]. Reductions in various parameters of healthcare resource utilization, particularly the number of sick leave days, were considerable, aligning with notable improvements in clinical outcomes. It is noteworthy that improvements in work productivity, as measured by presenteeism, absenteeism, and total work productivity impairment, have been demonstrated in previous observational studies, supporting the positive impact of adalimumab therapy on workforce productivity [85]. This holistic perspective, considering both clinical efficacy and economic benefits, underscores the nuanced evaluation required for the therapeutic use of adalimumab in managing AS and related conditions.

### 3.7 Adalimumab Safety Profile in Ankylosing Spondylitis

Wang *et al.* conducted a systematic review and meta-analysis [91] evaluating the safety and effectiveness of adalimumab treatment in individuals with ankylosing spondylitis (AS). The results indicated significantly lower rates of adverse events and injection-site reactions in the adalimumab group compared to the placebo group [1]. Furthermore, the study reported no noteworthy difference between the two groups concerning serious adverse events, infections, and drug discontinuation [1]. Another study by Burmester *et al.* [37] analyzed the long-term safety profile of adalimumab in various indications, including AS. The most frequently reported serious adverse events were serious infectious events, although the rate in AS patients did not exceed 0.2 per 100 patient-years [1]. There were no instances of active tuberculosis or severe opportunistic infections reported among patients diagnosed with ankylosing spondylitis (AS) [1]. The incidence rates of other adverse events, such as new-onset or worsening psoriasis, demyelinating disorders, lupus-like syndrome, congestive heart failure, and malignancies, were generally low across all indications [1]. Furthermore, the number of deaths observed in patients treated with adalimumab was lower than expected in certain populations [1]. These findings suggest a favorable safety profile for adalimumab in the treatment of AS.

## 4 DISCUSSION

The comprehensive evaluation of adalimumab's efficacy in the treatment of ankylosing spondylitis (AS) provided by this study unfolds a nuanced understanding that extends beyond the immediate scope of the investigation. By contextualizing these findings within the landscape of existing research and theoretical frameworks, a compelling narrative emerges, reinforcing the robustness of adalimumab as a therapeutic intervention for AS.

The congruence between the results of this study and prior research amplifies the consistent narrative of adalimumab's effectiveness in mitigating disease activity, enhancing physical function, and quelling inflammatory markers in individuals with AS. These collective findings substantiate the hypothesis that adalimumab, functioning as a tumor necrosis factor (TNF) inhibitor, assumes a pivotal role in orchestrating immune responses and alleviating the multifaceted symptoms associated with AS. The observed improvements across disease activity, physical function, and inflammatory markers not only validate the efficacy of adalimumab but also underscore its multifaceted impact on the pathophysiological processes underpinning AS.

The therapeutic potential illuminated by this study reverberates beyond the confines of its immediate focus, holding broader implications for the overarching management of AS. Adalimumab's demonstrated efficacy, particularly in reducing disease activity and enhancing physical function, transcends mere symptomatic relief, permeating into the realms of patient outcomes and overall quality of life. This suggests that the integration of adalimumab into the therapeutic arsenal for AS could yield profound benefits in the holistic care of affected individuals.

As these findings contribute to the accumulating body of evidence, the endorsement of adalimumab as an initial therapeutic option gains further strength. In the context of clinical practice, this burgeoning evidence base guides physicians in their treatment decisions, offering a roadmap to optimize patient care and improve treatment outcomes. The implications extend beyond the academic realm, permeating into the day-to-day decisions made by clinicians, ultimately shaping the landscape of AS management.

Looking ahead, future research trajectories should be meticulously charted to unravel the long-term efficacy and safety profile of adalimumab in AS. Prolonged studies meticulously assessing the durability of treatment response and scrutinizing potential risks associated with extended adalimumab use are indispensable for shaping evidence-based clinical guidelines. Beyond longevity, alternative treatment regimens, identification of predictors of treatment response, and exploration of potential combination therapies beckon attention. A concerted effort in these domains holds the key to optimizing AS management, enhancing treatment strategies, and ultimately elevating patient outcomes.

This study serves as a cornerstone, providing compelling evidence supporting the efficacy of adalimumab in the intricate landscape of ankylosing spondylitis. The congruence with prior research reinforces the therapeutic potential of adalimumab in decreasing disease activity and improving physical function among individuals with AS. These results, transcending the confines of academia, advocate for the consideration of adalimumab as a primary, first-line treatment option. However, the journey does not end here; further research is crucial to explore the intricate details of long-term effects and to refine treatment strategies, ensuring sustained progress in the quest to optimize patient care for individuals grappling with AS.

## CONCLUSION

This systematic review provides robust evidence supporting the effectiveness and safety of adalimumab in the treatment of ankylosing spondylitis (AS). The analysis of randomized controlled trials demonstrated that adalimumab significantly reduced disease activity, improved physical function, and lowered inflammatory markers, such as C-reactive protein and erythrocyte sedimentation rate, compared to placebo. Furthermore, the safety profile of adalimumab was favorable, with no significant increase in adverse events compared to placebo. This indicates that adalimumab is well-tolerated and can be considered a reliable treatment option for AS patients. The findings of this review support the use of adalimumab as a first-line therapy for AS, emphasizing its efficacy in managing symptoms and improving the overall quality of life for patients. The multifaceted benefits of adalimumab, encompassing clinical efficacy, reduced healthcare resource utilization, and improved work productivity, underscore its role as a valuable therapeutic option in the holistic management of AS. The integration of both clinical and economic considerations in the evaluation process ensures a comprehensive understanding of the impact of adalimumab, facilitating informed decision-making for healthcare professionals and policymakers alike. Clinicians and healthcare providers can confidently consider adalimumab as an effective and well-tolerated treatment option for patients with AS. The results of this study contribute to the growing body of evidence supporting the use of adalimumab in the management of this chronic inflammatory condition, providing hope for improved outcomes and enhanced quality of life for individuals living with AS.

## Figures and Tables

**Table 1:** Characteristics of the main randomized controlled trials.

**Clinical Trial**	**Number**	**Gender**	**Age**	**Intervention**	**Treatment Duration**	**Primary Outcomes**	**Adverse Events**
Revicki *et al.* [1]	A: 208	A: M 157; F 51	A: 41.7	Adalimumab40 mgs.c. eow	24	ASAS20, ASAS40, BASDAI, SF-36	NA
	P: 107	P: M 79; F 28	P: 43.4				
Huang *et al.* [55]	A: 229	A: M 185; F 44	A: 30.1	As above	12	ASAS20, ASAS40, BASDAI50, CRP, HAQ-S, SF-36	AE, serious AE, drug discontinuation, infection, serious infections
	P: 115	P: M 95; F 20	P: 29.6				
Sieper *et al.* [60]	A: 91	A: M 44; F 47	A: 37	As above	12	ASAS20, ASDAS,BASDAI, BASDAI50,SF-36, HAQ-S	AE, serious AE, drug discontinuation, infection, serious infections
	P: 94	P: M 40; F 54	P: 38.4				
Davis *et al.* [91]	A: 208	A: M 157; F 51	A: 41.7	As above	24	ASAS20, ASAS40, BASDAI, ASQoL, SF-36	NA
	P: 107	P: M 79; F 28	P: 43.4				
Hu *et al.* [91]	A: 26	A: M 24; F 2	A: 28.2	As above	24	BASDAI, BASFI, CRP, ASDAS	NA
	P: 20	P: M 20; F 0	P: 27.4				
van der Heijde *et al.* [91]	A: 208	A: M 236; F 79	A: 42.2	As above	12/24 rescue	BASFI, BASDAI, SF-36, ASQoL	AE, serious AE, drug discontinuation, infection, serious infections
	P: 107	P: M 219; F 69	P: 42.4				

**Abbreviations: **A, adalimumab group; P, placebo group; M, male; F, female; s.c., subcutaneously; eow, every other week; ASAS, Assessment in AS International Working Group; BASDAI, Bath Ankylosing Spondylitis Disease Activity Index; CRP, C-reactive protein; SF-36, Short Form 36; BASFI, Bath Ankylosing Spondylitis Functional Index; HAQ-S, Health Assessment Questionnaire modified for Spondyloarthropathies; ASDAS, Ankylosing Spondylitis Disease Activity Score; ASQoL, Ankylosing Spondylitis Quality of Life questionnaire; AE, adverse events; NA, not applicable.

## Data Availability

The data and supportive information are available within the article.

## LIST OF ABBREVIATIONS

AS: Ankylosing Spondylitis
THA: Total Hip Arthroplasty
TNF: Tumor Necrosis Factor

## STANDARDS OF REPORTING

PRISMA guidelines and methodology were followed.

## SUPPLEMENTARY MATERIAL

PRISMA checklist is available as supplementary material on the publisher’s website along with the published article.
